# Uniconazole Augments Abscisic Acid in Promoting Somatic Embryogenesis in Cotton (*Gossypium hirsutum* L.)

**DOI:** 10.3389/fpls.2022.865778

**Published:** 2022-04-04

**Authors:** Yanli Chen, Hongxia Yu, Ye Wang, Fuguang Li, Yadi Xing, Xiaoyang Ge

**Affiliations:** ^1^Zhengzhou Research Base, State Key Laboratory of Cotton Biology, School of Agricultural Sciences, Zhengzhou University, Zhengzhou, China; ^2^State Key Laboratory of Cotton Biology, Institute of Cotton Research, Chinese Academy of Agricultural Sciences, Anyang, China; ^3^College of Plant Science and Technology of Huazhong Agricultural University, Wuhan, China

**Keywords:** cotton, uniconazole, IAA, abscisic acid, somatic embryogenesis

## Abstract

During somatic embryogenesis (SE), somatic cells initiate embryogenic development under appropriate conditions. Uniconazole, a plant growth regulator, was found to inhibit the proliferation of callus but promoted the conversion of callus into an embryogenic callus (EC) in cotton. The supplementation of uniconazole in the culture medium significantly suppressed the endogenous auxin [indole acetic acid (IAA)] level in callus tissues in both the callus initiation and proliferation stage but enhanced the abscisic acid (ABA) level only in the callus proliferation stage. Exogenous ABA and uniconazole showed cooperative effects on promoting the differentiation rate of callus into EC. These findings were verified by RNA-seq analysis, which elucidated that the genes involved in the IAA biosynthesis, metabolism, and signaling, and ABA metabolism pathways were regulated by uniconazole during the callus development and SE. Overall, the results suggest that uniconazole could modulate callus proliferation and callus differentiation rate by regulating the endogenous levels of IAA and ABA.

## Introduction

Plant cells exhibit remarkable totipotency manifested by plant regeneration from single somatic cells (Ikeuchi et al., 2016). Somatic embryogenesis (SE) is a notable elucidation of plant cell totipotency in which somatic cells undergo dedifferentiation and redifferentiation to generate embryogenic cells, leading to the formation of somatic embryos. Somatic embryo formation resembles the zygotic embryogenic process, including globular embryo, torpedo embryo, and cotyledon embryo stages, and then generates new plants under appropriate induction conditions (Jin et al., 2014). SE is important not only for fundamental research but also for biotechnological applications.

Auxin regulation of SE has been demonstrated in model systems (Kim et al., 2000; Komamine et al., 2005). It is commonly observed that auxin is distributed in the form of gradients in the explants in tissue culture *via* local biosynthesis, degradation, and polar transport (Vanneste and Friml, 2009; Wabnik et al., 2013). The formation of an auxin gradient in explants is essential for SE (Su et al., 2009), which regulates the expression of a suite of genes related to cell division and cell differentiation, such as *GH3s, PINs*, indole acetic acids (*IAAs*), *SAURs*, and *ARFs* (Fischer and Neuhaus, 1996; Yang and Zhang, 2010). In cotton (*Gossypium hirsutum*), as many as 86 genes related to auxin were differentially expressed in the process of SE (Yang et al., 2012). Transcriptome profiling revealed that genes involved in auxin biosynthesis and signal transduction pathways were differentially expressed during SE in cotton (Xu et al., 2013; Cao et al., 2017). Furthermore, the genome-wide analysis found that auxin early response genes were co-expressed with some SE-related genes and played important regulatory roles in the key step of embryogenic competence acquisition in cotton (Sun et al., 2019).

Apart from auxin, abscisic acid (ABA) has also been found to play an important role in SE, whereby an appropriate concentration of exogenous ABA could promote SE and improve the quality of somatic embryos (Kiyosue et al., 1991). It has also been reported that exogenous ABA could suppress the formation of non-embryogenic callus (EC) (Rajasekaran et al., 1987) and increase the number of plantlets generated from SE (Qureshi et al., 1989). Corroborating results were provided by biochemical analysis of endogenous ABA that showed substantially higher levels in EC and somatic embryos than in non-EC cells (Kiyosue et al., 1992). Endogenous ABA in plants is coordinately controlled by its biosynthesis, transportation, and catabolic inactivation in response to various environmental factors. In particular, ABA catabolism is mainly controlled by ABA 8′-hydroxylase that is a cytochrome P450 enzyme catalyzing the C8′-hydroxylation of ABA leading to the formations of 8′-hydroxy-ABA (8′OH-ABA) and phaseic acid (PA) that has much lower hormonal activity than ABA. Numerous studies in model plants have demonstrated that ABA 8′-hydroxylase plays an important role in determining threshold levels of ABA, affecting numerous aspects of plant growth and abiotic stress responses (Umezawa et al., 2006). Attempts have been made to control plant endogenous ABA levels by manipulating this highly controlled system, especially ABA catabolism in order to explore ABA functionalities and their underlying mechanisms (Huang et al., 2007).

A plant growth-regulator known as uniconazole was identified as a potent inhibitor of ABA 8′-hydroxylase (Saito et al., 2006). Uniconazole is a member of the triazole family that is known to bind to the active sites of cytochrome P450 enzymes by both coordinating to the heme-iron atom and interacting with neighboring amino acid residues (Saito et al., 2006). Despite the fact that it has the side effect of inhibiting the biosynthesis of gibberellic acid (GA) and brassinosteroid (BR) (Min et al., 1999), uniconazole can protect plants from various stresses, in addition to various other functions, such as enhancing the levels of carotenoid and chlorophyll (Fletcher and Hofstra, 1990), promoting the accumulation of starch (Liu et al., 2015), improving primary root elongation and delay bolting in *Arabidopsis*, controlling the flowering, enhancing fruit setting (Wei et al., 2017), elevating yield components in crop plants (Zhou and Ye, 1996; Ahmad et al., 2018), and changing the content of endogenous hormones (Zhang et al., 2007).

*Gossypium hirsutum* is one of the most important economic crops, which provides most of the textile fibers in the world. Cotton is also one of the earliest genetically modified crops that have gained great commercial success. However, further development is constrained by the intrinsic feature of cotton that is recalcitrant to SE and regeneration through tissue culture in most elite cultivars, in addition to the long culture time and low efficiency in forming EC (Xu et al., 2015). Proteomic studies have demonstrated that stress response, hormone homeostasis, respiration, and photosynthesis influence the formation and development of somatic embryos in cotton tissue culture (Ge et al., 2015; Zhou et al., 2016). Compared with studies on somatic embryos, there are scant reports about the factors that determine the conversion of non-ECs (NECs) to ECs, which is a prerequisite for the formation of the somatic embryo and subsequent plant regeneration. In this study, four different cotton germplasms, representing variable levels of callus responsiveness to the induction of SE, were used to investigate the potential functional role of uniconazole in promoting SE in cotton. Furthermore, we have also explored the regulatory mechanism underpinning the uniconazole effects on SE, which were coordinately modulated by the interaction of endogenous auxin and ABA.

## Materials and Methods

### Plant Materials and Culture Conditions

In this study, four *G. hirsutum* varieties, including TM-1, 99668, J14, and ZM24, were chosen, representing null, low, and high conversion rates of callus into EC, respectively. Seeds were surface sterilized with 70% ethanol for 1 min, rinsed at least three times with sterile water, and imbibed in 30% (v/v) H_2_O_2_ for 24 h. The sterilized seeds were germinated on MS medium (Murashige and Skoog, 1962) in the dark at 28°C for 5 days. Hypocotyls were dissected from the seedlings and cut into 5–7 mm segments and cultivated on callus-induction medium as specified in the “Uniconazole and ABA treatments during callus induction and differentiation stages” section (Zheng et al., 2014).

### Uniconazole and Abscisic Acid Treatments During Callus Induction and Differentiation Stages

To determine the effect of uniconazole on callus growth and the conversion rate of callus into EC, the hypocotyl explants were cultured on callus-induction medium (MS salts, B5 vitamins, 0.10 mg/L 3-IAA, 0.10 mg/L kinetin, 0.10 mg/L 2,4-D, 25 g/L glucose, and 2 g/L Gelrite^®^ at pH 5.8) supplemented with 7 μM uniconazole under standard conditions (28°C and 14 h L/h D) for 2 months. To investigate the interactive effects of exogenous 0.04 μM ABA and 7 μM uniconazole (Sigma. Darmstadt, Germany) on SE, the explants of ZM24 were transferred onto freshly prepared callus-induction medium supplemented with appropriate amounts of ABA and uniconazole.

### Vector Construction and Transformation

Synthetic auxin response element (DR5) was cloned into the vector pCambia2300 replacing 35 promoters and fused to a GFP reporter gene, constructing the vector DR5::GFP. The vector was transformed into *G. hirsutum* ZM24 plants *via Agrobacterium tumefaciens* (LB4404) as described earlier (Zhan et al., 2021).

### Calculation of Callus Proliferation Rate and Embryonic Differentiation Rate

Callus proliferation rate (CPR) was calculated as the fold change in weight gained of explants at 15 and 25 days of induction as described by Wang et al. (2017). The embryonic differentiation rate (EDR) was calculated by the percentage of explants with embryonic callus at different time points on the callus-induction medium or those supplemented with uniconazole and ABA.

### Scanning Electron Observation

For scanning electron observation, callus was placed on a fixed plate with a conductive adhesive, prior to being photographed under a HITACHI SU3500 scanning electron microscope (Hitachi, Tokyo, Japan).

### Alkaline Phosphatase Staining

Alkaline phosphatase was used as the marker for identifying pluripotent stem cells (Štefková et al., 2015). Alkaline phosphatase staining was performed using the BCIP/NBT Alkaline Phosphatase Staining Kit (Beyotime Biotechnology, Shanghai, China) following the manufacturer’s instruction with modifications. The staining solution was added to a 10-ml centrifuge tube and incubated with NEC and EC for 30 min. Following the removal of the staining solution, the NEC and EC were washed with distilled water once before being transferred into absolute ethanol and photographed.

### Endogenous Indole Acetic Acid and Abscisic Acid Extraction and Quantification

After being ground to powder in liquid nitrogen, approximately 1 g of the samples were homogenized in 5 ml prechilled 80% methanol solution and vortexed for 90 h at 4°C. The extracts were then centrifuged at 12,000 *g* for 1 h, and the supernatant was collected and evaporated under the flow of nitrogen gas until being reduced to half of the original volume. The supernatant was purified using the C18 Sep-Pak cartridge that had been activated with 3 ml 100% methanol and preequilibrated with the formic acid solution, and then, the supernatant was dried under the flow of nitrogen gas. The dry residue was redissolved in 200 μl methanol, and the solution was filtrated with 0.22 μm syringe filter and 10 μl filter eluate was taken for the high-performance liquid chromatography (HPLC) analysis. Three biological replicates were performed.

### RNA-Seq Analysis

Hypocotyls of ZM24 that were cultured on the callus-induction medium supplemented with or without uniconazole were sampled for RNA-seq analysis. Samples were collected at 7, 15, and 30 days after the initiation of culture, which were used to construct the RNA libraries. RNA-seq, consisting of two biological replicates, was performed using Illumina HiSeq™ 2000 (BGI, Shenzhen, China). The raw reads were subjected to quality control and filtered into clean reads, prior to being mapped to the reference genome of *G. hirsutum* (Li et al., 2015). Gene expression was calculated by Fragments Per Kilobase of transcript per Million mapped reads (FPKM), and differentially expressed genes (DEGs) was identified by fold change ≥ 2 and false discovery rates (FDR) with adjusted *p*-value < 0.01 combining three biological replicates. The data are available in the NCBI SRA under the number PRJNA808630.

### RNA Isolation and Quantitative Real-Time PCR Analysis

The total RNA was isolated from cotton callus and leaves using the RNAprep Pure Plant Kit (Tiangen). First-strand cDNA was generated from the total RNA using the PrimeScript RT Reagent Kit (Takara). qRT-PCR was performed using SYBR Premix Ex Taq™ (Takara). *GhHISTONE* was used as an endogenous reference gene, and the relative expression level was calculated using the 2^–△△CT^ method as described previously (Xu et al., 2015). The gene-specific primers are listed in Supplementary Table 1.

### Statistical Analysis

All statistical analyses were based on at least three biological replicates, and all the values are displayed as the mean ± SD. Statistical analysis was determined using the *t*-test, and *p < 0.05 and **p < 0.01 were considered statistically significant.

## Results

### Uniconazole Inhibited Callus Proliferation Independent of Genotype

Seven days after spraying with 20 mg/L uniconazole, the growth of cotton seedlings was retarded as shown by significantly reduced plant height relative to the unsprayed control plants (Supplementary Figure 1A). The top and reciprocal second leaves were smaller, wrinkled, and with short petioles (Supplementary Figure 1B). The histological analysis of the longitudinal paraffin sections prepared from the reciprocal fourth petioles illustrated that the cell size, elongation in particular, of the cortex cell layer was conspicuously reduced in the uniconazole-treated seedlings relative to the control (Supplementary Figure 1C), suggesting the inhibition of cell elongation or growth rate on uniconazole treatment.

**FIGURE 1:**
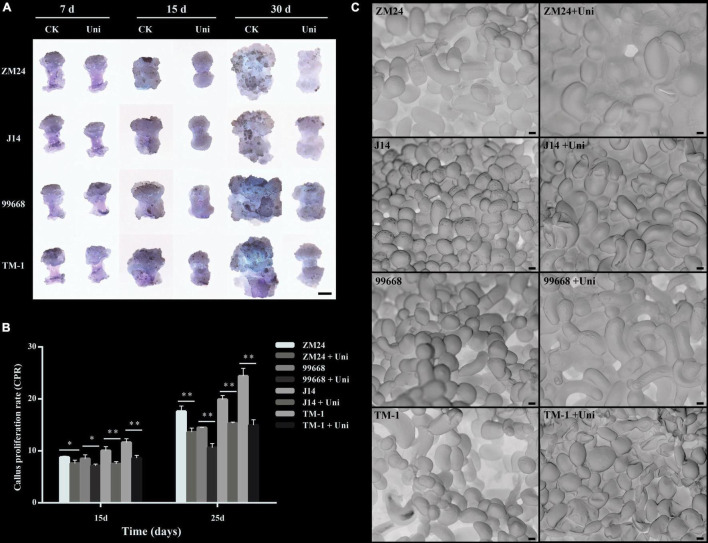
Effects of uniconazole on callus proliferation and callus cell morphology in *Gossypium hirsutum* cv ZM24, J14, 99,668, and TM-1. **(A)** The phenotype of callus after cultured for 7, 15, and 30 days on callus-induction medium without the supplementation of uniconazole (CK) and those with uniconazole (Uni). Scale bar = 5 mm. **(B)** The scanning electron microscopy images of callus cells of ZM24, J14, 99,668, and TM-1, at 30-day induction. Scale bar = 20 μm. **(C)** Callus proliferation rate (CPR) recorded after culturing for 15 and 25 days. The single asterisk indicates statistical significance at *p* < 0.05. The double asterisk indicates statistical significance at *p* < 0.01.

Given that the uniconazole spraying significantly affected the growth of cotton seedlings *via* repressing cell elongation, the question arises as to whether uniconazole could affect callus growth and SE in cotton tissue culture. To address such a query, callus derived from four *G. hirsutum* cultivars with the variable level of SE capability was subjected to uniconazole treatment by supplementing 7 μM uniconazole to callus-induction medium. This concentration of uniconazole was chosen in accordance with a preliminary gradient experiment that suggested 7 μM uniconazole as the optimal level for growth suppression (Supplementary Figure 2). Following 7-day incubation on callus-induction medium supplemented with uniconazole, the hypocotyl explants produced callus mass at their ends, without discernible variation between control and uniconazole treatment (Figure 1A), indicating that uniconazole may not exert a significant inhibition effect on cell growth during the callus initiation process. At 15 days after the initiation of culture, the callus in the uniconazole treatments appeared to be smaller than the control callus and exhibited abnormality in callus appearance showing compactness rather a smooth surface, which is in contrast to the well-developed control callus (Figure 1A). At 30 days in culture, calluses in the control explants were substantially larger in size relative to the uniconazole-treated explants (Figure 1A). The effect of uniconazole on cell growth was further quantified using the CPR in the callus proliferation stage, showing that uniconazole treatment resulted in significant reductions in the CPR at days 15 and 25 independent of the genotype (Figures 1A,B).

Given that uniconazole inhibited callus growth *via* decreasing the cell size, we then investigated if the regulation function of uniconazole is conserved in seedling and SE. A scanning electron microscope was used to facilitate the measurement of the callus cell size. Strikingly, it was the reduction in the number of callus cells rather than the cell size that could be attributable to relatively smaller callus in the uniconazole treatments relative to the untreated control (Figure 1C). Notably, the large callus cells increased in numbers under uniconazole treatment at a relatively fast pace than control, but the rate of cell division was reduced, resulting in lesser cell proliferation and gross reduction in the callus size as observed in the uniconazole treatments. In essence, the suppressing effect of uniconazole on growth is common between seedling and callus, but through a different route, negatively impacting cell size in the former and cell number in the latter.

### Uniconazole Regulates Cell Fate Specification During Somatic Embryogenesis

**FIGURE 2:**
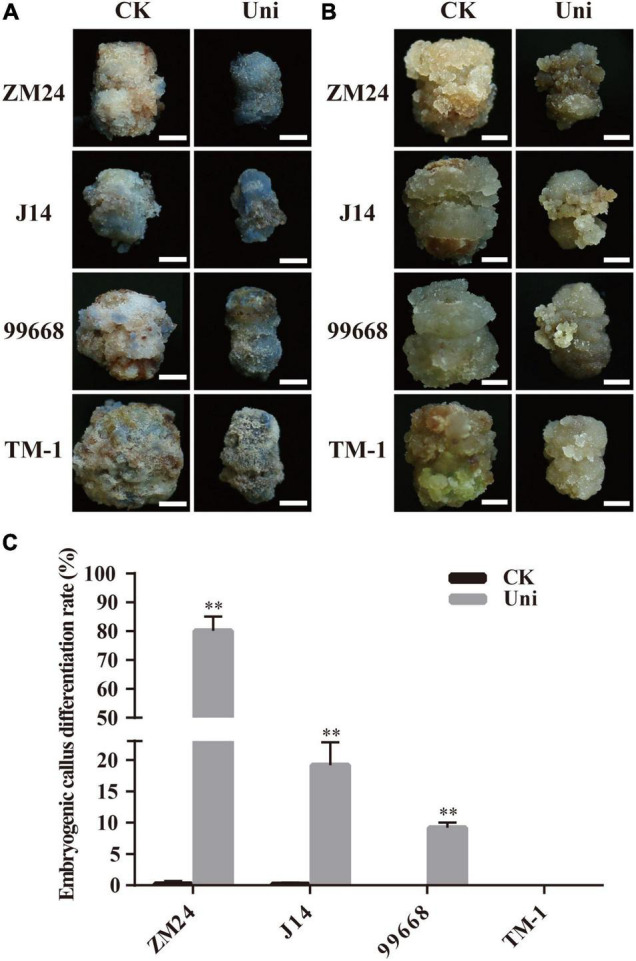
Uniconazole positively promotes the conversion of callus into an embryogenic callus (EC) during cotton tissue culture. **(A)** Alkaline phosphatase staining of callus in ZM24, J14, 99,668, and TM-1 at 30 days (d) in culture. Scale bar = 5 mm. **(B)** Callus appearance of ZM24, J14, 99,668, and TM-1 at 40-day induction under CK or uniconazole (Uni) treatment. Scale bar = 5 mm. **(C)** EC differentiation rate of ZM24, J14, 99,668, and TM-1 at 80-day induction under CK or Uni treatment. The double asterisk indicates statistical significance at *p* < 0.01.

Alkaline phosphatase shows lower activity in differentiated cells than pluripotent stem cells; therefore, it could be used to evaluate cell pluripotent properties (Zhao et al., 2009). To investigate whether uniconazole could impact the capacity of callus cells for differentiation into stem cells, the explants of ZM24, J14, 99668, and TM-1 were maintained on the callus-induction medium with or without uniconazole and were sampled for alkaline phosphatase staining at 30 days. In contrast to the rather weak and sporadic bluish staining in a very small fraction of callus on the control medium, distinct blue color was observed in a large fraction of callus derived from ZM24, J14, and 99,668 on the medium supplemented with uniconazole. It was noted that TM-1 callus was only slightly stained under uniconazole treatment (Figure 2A), without conspicuous variation between control and uniconazole treatment.

To detect the relationship of alkaline phosphatase activity and pluripotent stem cells, a part of callus was continuously cultured to induce EC until the 40-day culture. A fraction of EC in ZM24, J14, and 99,668 was obtained under uniconazole treatment, but only an enlarged callus mass was present on the control plates (Figure 2B). In line with the weak activity of alkaline phosphatase, EC was not obtained, except a small fraction of callus displaying EC-like features after uniconazole treatment in TM-1. Therefore, it is evident that uniconazole could promote cell fate specification in the genotypes with EC regeneration capacity, but not in those without such an ability.

For further quantification of the SE-promoting efficiency of uniconazole, the EDR representing the transition rate of callus into EC was then measured in the aforementioned genotypes at 60 days. It is overt that EDR was significantly increased under uniconazole treatment, whereby EDR accounted for 80.12% in ZM24, 19.17% in J14, and 9.19% in 99,668 under uniconazole treatment, which was in sharp contrast to 0.25% in ZM24, 0.17% in J14, and null in 99,668 on the control explants (Figure 2C). As anticipated, no EC was obtained in TM-1 with or without uniconazole (Figures 2B,C). But for the remaining three genotypes, unlike a small proportion of callus that could form EC without the supplementation of uniconazole, most of the calli under uniconazole treatment could differentiate into EC (Supplementary Figure 3). Overall, as alkaline phosphatase activity is positively related to EDR, it could be used as a practical indicator for the potential of pluripotent stem cell acquirement in cotton callus.

### Uniconazole Modulates Endogenous Indole Acetic Acid and Abscisic Acid Levels During Somatic Embryogenesis

**FIGURE 3:**
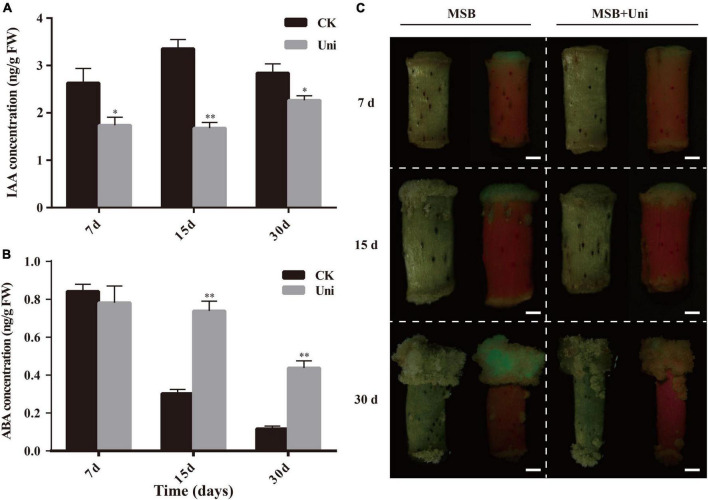
Effects of exogenous uniconazole on the plant hormone levels at different stages of callus in ZM24. **(A)** DR5-GFP expression in calli of ZM24 transformed plants at 7 days (d), 15- and 30-day induction on Murashige and Skoog Basal (MSB) medium or MSB medium-plus uniconazole (Uni), scale bar = 1 mm. **(B)** Indole acetic acid (IAA) concentration in callus during induction and proliferation stages at 7, 15, and 30 days of cultivation under CK or uniconazole (Uni). **(C)** Abscisic acid (ABA) concentrations at callus induction and proliferation stage. The single asterisk indicates statistical significance at *p* < 0.05. The double asterisk indicates statistical significance at *p* < 0.01.

It has been reported that uniconazole could change the content of endogenous hormones (Izumi et al., 1988). Considering that auxin and ABA play an important role in SE (Yang et al., 2012; Jin et al., 2014), the concentrations of IAA and ABA in ZM24 callus at 7-, 15-, and 30-day postinduction were measured by HPLC-MS. As described in Figure 3A, the IAA concentration of callus grown on CIM supplemented with uniconazole was decreased significantly compared with the control at all-time points. It is suggested that the reduction of the auxin content in callus was a pivotal factor for inhibiting callus growth on uniconazole treatment. Different from the downward trend of auxin in uniconazole treated callus, the change in ABA concentration is more complex. After 7-day induction in the callus initiation stage, there was a slight decrease, yet not statistically significant, in the ABA content in the uniconazole treatment relative to control. However, at 15 and 30 days at the callus proliferation stage, the ABA contents in uniconazole treated callus rose significantly relative to control (Figure 3B). The reduction in the auxin content coupled with a rise in the ABA level may act in concert in inhibiting callus proliferation on uniconazole treatment.

### Auxin Distribution in Cotton Callus Was Affected by Uniconazole

*DR5*, a highly active synthetic auxin response element (*AuxRE*), displays the distribution of auxin in tissues (Ulmasov et al., 1997). Transgenic cotton containing the auxin-responsive reporter DR5::GFP was used for the assessment of the spatial pattern of auxin distribution in cotton callus at both callus initiation and proliferation stages, using the GFP fluorescence signal. As shown in Figure 3, uniconazole treatment reduced the IAA content in the callus and influenced the fate of the callus; therefore, the auxin content and its distribution are both important for cell growth and development. Consistent with the decrease in the endogenous auxin level, the GFP fluorescence intensity of the callus under the uniconazole treatment was overtly weaker than control in both callus initiation and proliferation stages. At 7 days, the initiation of callus formation was observed at one end of a hypocotyl cultivated on the Murashige and Skoog Basal (MSB) medium, without discernible callus formation by naked eyes at the other end on uniconazole treatment (Figure 3C). Clear green fluorescence was observed in the callus formed on hypocotyl in the MSB medium (control), which was in contrast to the uniconazole-treated hypocotyls that did not show visible GFP. Likewise, a stronger and more widely distributed GFP fluorescence signal was exhibited in the control callus relative to uniconazole-treated callus at 15 and 30 days, suggesting the inhibitory role of uniconazole in callus proliferation *via* reducing the auxin level and its transport (Figure 3C).

### Abscisic Acid and Uniconazole Cooperatively Promoted the Transition of the Callus Cell Into the Embryogenic Cell

**FIGURE 4:**
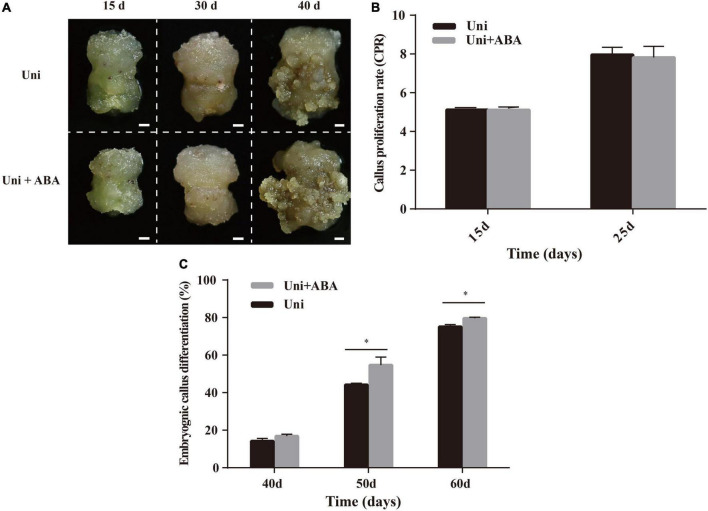
Effects of exogenous uniconazole (Uni) and ABA on the conversion of callus to EC in ZM24. **(A)** Status of callus and EC after 15-, 30-, and 40-day induction on callus induction medium containing Uni or both Uni and ABA. Scale bar = 2 mm. **(B)** The CPR of ZM24 after induction for 15 and 25 days. **(C)** The embryonic differentiation rate (EDR) of ZM24 after induction for 40, 50, and 60 days. The single asterisk indicates statistical significance at *p* < 0.05.

The increase in the ABA content in uniconazole-treated callus (Figure 3) was concomitant with the promotion in pluripotent stem cell and EC acquirement, suggesting ABA and uniconazole acting in concert to determine cell fate transition. To facilitate the investigation on such a premise, explants of ZM24 were cultured on callus induction media containing uniconazole with or without (control) exogenous 0.04 μM ABA. The concentration of ABA was chosen according to preliminary gradient experiments (Supplementary Figure 4). Callus presented the same texture and color regardless of the ABA supplement at 15 and 30 days (Figure 4A). Quantitative analysis on callus growth using the CPR did not show significant variation between uniconazole and uniconazole + ABA treatment (Figure 4B). However, the analyses of the EC phenotype and EDR on the cultures between 40 and 60 days appeared to be very different. As shown in Figure 4A, at 40 days, embryonic cell mass grown on the medium supplemented with both ABA and uniconazole was visibly larger than those treated with ABA alone. Such an observation was supported by the quantitative analysis on EC differentiation rate that showed significant increments compared with the control at 40, 50, and 60 days (Figure 4C). By virtue of these data, it is conceivable that ABA and uniconazole displayed additive effects on promoting cell fate transition from callus cell into EC but not on callus proliferation.

### Genes Involved in Auxin, Abscisic Acid Pathway, and Somatic Embryogenesis Were Differentially Expressed on Uniconazole Treatment

To explore the molecular mechanism underpinning the uniconazole regulation on SE in cotton, genome-wide transcript profiling of callus with or without uniconazole treatment was performed. After quality control and filtering, approximately 50 million clean reads were generated in each library. Approximately 93% of reads were mapped to *G. hirsutum* TM-1 genome sequences (Li et al., 2015), and approximately 90% were mapped to gene sequences (Supplementary Table 2).

**FIGURE 5:**
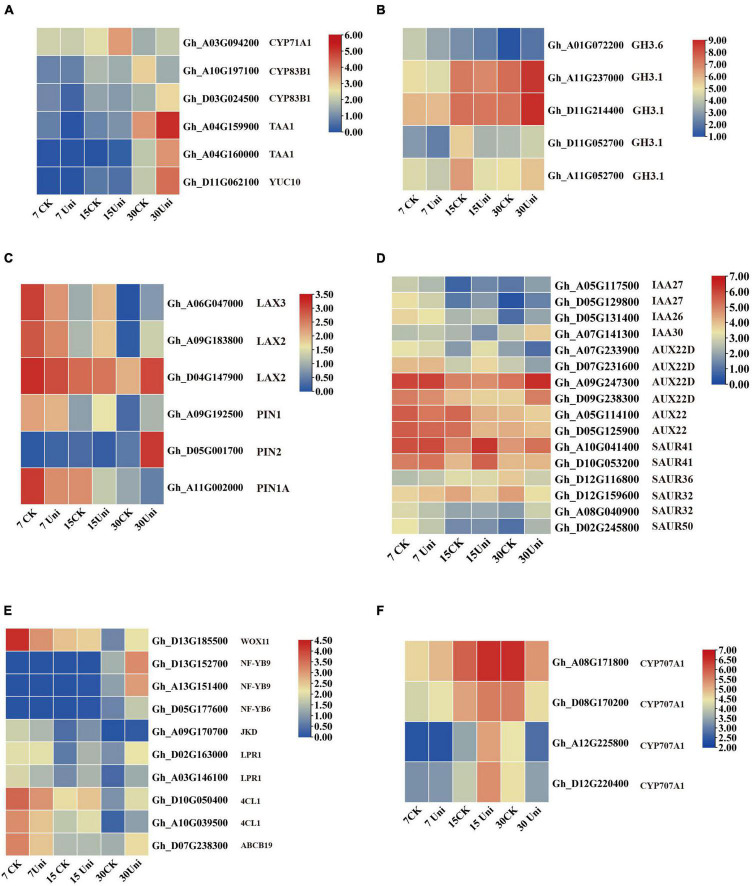
Genes related to auxin, ABA, and somatic embryogenesis (SE) were differentially expressed in cultured callus with or without the supplementation of uniconazole. **(A–D)** Heat map of differentially expressed genes (DEGs) involved in auxin biosynthesis, metabolism, and signaling pathway, shown as Log2 values. **(E)** Heat map of DEGs involved in ABA metabolism pathway, shown as Log2 values. **(F)** Heat map of DEGs involved in transcription factors for SE, shown as Log2 values.

Compared with control, 47 genes related to auxin biosynthesis, transport, and signaling pathway were differentially expressed in the callus under uniconazole treatment relative to the untreated control. Six auxin biosynthesis transcripts, encoding cytochrome P450 (CYP71A1 and CYP83B1), L-tryptophan pyruvate aminotransferase 1 (TAA1), and indole-3-pyruvate monooxygenase (YUCCA10), showed a sophisticated expression pattern at different cultural stages. Compared with control, most of the auxin biosynthesis-related transcripts exhibited lower expression levels at 7 and 15 days but upregulated at 30 days on uniconazole treatment (Figure 5A and Supplementary Table 3), corroborating the biochemical analysis of endogenous auxin analysis. Five indole-3-acetic acid-amido synthetase transcripts (GH3.1 and GH3.6) showed a relatively higher expression level than control, except for the GH3.6 transcript (Gh_–_A01G072200) that maintained its lower expression level at all three time points. Compared with control, the expressions of *GH3* genes were downregulated at 7 and 15 days and before their upregulation at 30 days on uniconazole treatment (Figure 5B). GH3 catalyzed the synthesis of IAA-amino acid conjugates to cope with the presence of excess auxin, and increased auxin level under uniconazole may need high expressed GH3 at 30 days. The auxin transport-associated genes, including three *LAX* genes and three *PIN* genes, were differentially expressed. Regardless of uniconazole treatment or not, the expression levels of *LAX* and *PIN* genes showed a consistent decrease along with the progress of uniconazole treatment (Figure 5C), indicating the weakening of auxin polar transport during callus growth. Furthermore, compared with control, most of the *LAX* and *PIN* genes, with the exception of *PIN1A*, were downregulated at 7 days, prior to their upregulation by uniconazole treatment at 15 and 30 days (Figure 5C). The differential expression of auxin transport genes may be well attributed to the differential auxin level in both the control and uniconazole treated callus.

A number of auxin-responsive protein genes were differentially expressed. Except for IAA30, two IAA27 members and one IAA26 were downregulated at 7 days before being upregulated at 30 days with uniconazole treatment. Most of the *Aux* genes, with the exception of two AUX22D genes, were highly expressed at all the three callus stages in the control. However, their gene expression patterns were substantially altered by uniconazole treatment. The response of six *SAUR* genes to uniconazole treatment was variable. SAUR41 showed a high level of expression at all the time points, which was further upregulated by uniconazole treatment. In contrast, other *SAUR* members, such as *SAUR32* and *SAUR50*, showed rather a low expression throughout. *SAUR32* was induced by uniconazole treatment at 7 days but downregulated at 15 and 30 days, while the opposite was true for *SAUR50* (Figure 5D). Taken together, it appears that auxin biosynthesis, transport, and signaling pathway may have participated in the interactive modulation with uniconazole for callus growth and differentiation.

Four transcripts corresponding to the key enzyme ABA catabolism, ABA 8′-hydroxylase CYP707A1, including Gh_–_A08G171800, Gh_–_D08G170200, Gh_–_A12G225800, and Gh_–_D12G220400, were all upregulated by uniconazole treatment at 7 and 15 days, before being downregulated at 30 days (Figure 5F). ABA catabolism might need to be appropriately upregulated *via* negative feedback regulation from 7 to 15 days in order to mitigate the adversity stress stem from excessive endogenous ABA levels in the callus on uniconazole treatment, which are detrimental to cell development. From 15 to 30 days, the concentration of ABA decreased in callus with or without uniconazole treatment. Unlike the control, the high level of endogenous ABA as the result of the downregulation of ABA catabolism following uniconazole treatment is likely favorable for cell fate transition from callus cell to embryogenic cell.

Ten SE-associated transcription factors were differentially expressed at 7, 15, and 30 days. WUSCHEL-related homeobox transcript, *WOX11*, displayed high expression at 7 days in control, which was downregulated at the same time point in uniconazole treatment (Figure 5E). This is congruent with a previous report that WOX11 positively regulates callus initiation and proliferation (Liu et al., 2014), as the reduced expression of *WOX11* on uniconazole may hinder callus growth. Other transcripts, including *JACKDAW* (*JKD*), *LOW PHOSPHATE ROOT1* (*LPR1*), *ABCB19*, and *4-COUMARATE*: *COA LIGASE1* (*4CL1*), all exhibited similar expression patterns with *WOX11*, whereby they were highly expressed at 7 days, but downregulated on the uniconazole treatment (Figure 5E). Three nuclear transcription factor Y subunit transcripts encoding NF-YB9 (Gh_–_D13G152700 and Gh_–_A13G151400) and NF-YB6 (Gh_–_D05G177600) were not expressed at 7 and 15 days but clearly upregulated expression at 30 days, showing significant induction in uniconazole treated callus relative to control. It is particularly intriguing to explore whether the increased expression of *NF-YB* genes in the callus at 30 days could promote the cell fate transition from callus cell to embryogenic cell, which warrants further study. Furthermore, qRT-PCR was used to validate the RNA-seq results and confirmed that some auxin-, ABA-, and SE-related genes were differentially expressed in the callus under uniconazole treatment (Supplementary Figure 5).

## Discussion

### Uniconazole Inhibits Callus Proliferation and Promotes Embryogenic Cell Acquirement

Numerous studies have shown that uniconazole plays an important role in SE in several plant species, accentuating somatic embryo development. For example, uniconazole significantly improved the production of somatic embryos and their further development to plantlets in asparagus (Li and Wolyn, 1995). In geranium and carrots, the number of somatic embryos was increased by uniconazole treatments in the somatic embryo growth and differentiation period (Hutchinson et al., 1997; Tokuji and Kuriyama, 2003). In contrast, less is known about the effect of uniconazole on callus growth and embryonic callus production. It was reported that uniconazole was able to promote callus induction from maize immature embryos and rice mature embryos, improving callus differentiation in rice but not in maize (Fu et al., 2005; Shan and Zou, 2014). It appears that the functional role of uniconazole in promoting callus differentiation and transition to somatic embryos may vary from plant species to plant species and invokes exploration in individual crops before using it as an important research tool in assisting plant regeneration through tissue culture and genetic modification. To our knowledge, no study has hitherto reported the impact of uniconazole on SE in cotton. In this study, we uncovered the role of uniconazole on SE in different cotton varieties (cultivars with low or high regeneration ability and a recalcitrant variety TM-1). Uniconazole inhibited callus proliferation independent of genotypes (Figure 1), whereby decreasing cell numbers resulted in smaller callus in size. The ratio of the callus conversion into embryonic callus was overtly improved along with the increase in the size of embryogenic cell mass in ZM24, J14, and 99,668 lines, but in TM-1, only the texture of callus was changed on uniconazole treatment (Figure 2). It is conceivable that embryogenic cell specification is dependent on multifaceted factors, among which uniconazole plays an assistive role instead of a decisive factor in promoting cotton SE. In essence, uniconazole inhibited callus growth in a genotype-independent manner and in a facilitating role in enhancing EC differentiation rate in the genotypes with regeneration ability.

### Uniconazole Interacts With Plant Hormones to Modulate Cell Fate Transition

Callus and embryonic callus induction are essential to cotton SE, which is a prerequisite for producing transgenic cotton. The molecular mechanisms of callus induction and differentiation have been revealed, where auxin, cytokinin, and wound signal played key roles in the reacquisition of embryonic or meristematic fate (Ikeuchi et al., 2013). Roles of uniconazole in regulating plant hormones have been demonstrated in rice, winter rape, soybean, and bioenergy crop duckweed (*Landoltia punctata*) (Izumi et al., 1988; Zhou and Leul, 1998; Zhang et al., 2007; Liu et al., 2015). The effect of uniconazole on the endogenous IAA level in callus was variable depending on plant species. In winter rape, the endogenous IAA level was decreased following foliar application of uniconazole (Zhou and Leul, 1998, 1999), while in rice seedlings, IAA was not altered on uniconazole treatment (Izumi et al., 1988). In our research, endogenous IAA concentration was reduced significantly from the early stage of callus initiation to the formation of non-EC after uniconazole treatment (Figure 3A). Concomitant inhibition of GFP fluorescence was observed in uniconazole-treated callus (Figure 3C). These results indicated that uniconazole regulates cell fate transition by reducing the content of auxin and disrupting its polar distribution, low auxin level may repress the callus over-proliferated growth and promote EC acquirement, and congruent with the previous report that the endogenous auxin level was significantly lower in EC and somatic embryos than those without embryogenic potential (Yang et al., 2012).

Uniconazole has been shown to regulate the endogenous concentration of ABA by inhibiting the activity of the ABA catabolism enzyme (Todoroki et al., 2008, 2012). Uniconazole treatment gave rise to an increase in the ABA level in plants (Zhou and Leul, 1999; Zhang et al., 2007; Li et al., 2015). In this study, the callus contained significantly higher endogenous ABA levels following uniconazole treatment (Figure 3B), supporting the premise that enhancement in the ABA level could facilitate EC production, which is validated by the cooperative effects of ABA and uniconazole on EC differentiation.

### Uniconazole Modulates the Expression of Transcription Factors Involved in Somatic Embryogenesis

**FIGURE 6:**
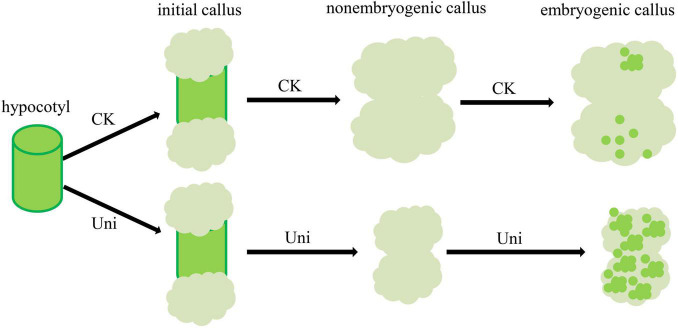
A proposed model for the role of uniconazole during SE in cotton. Uniconazole inhibits the proliferation of callus and promotes EC formation.

The importance of transcription factors to the process of SE has been demonstrated in an eclectic list of studies. *WUSCHEL ELATED HOMEOBOX 11* (*WOX11*) is involved in the first step of cell fate transition in the *de novo* root organogenesis process, which shares a similar mechanism with callus formation (Liu et al., 2014). LEAFY COTYLEDON1 (LEC1) can maintain embryonic cell fate at an early development stage in *Arabidopsis* (Lotan et al., 1998), by regulating the key genes involved in cellular differentiation and embryo development (Kwong et al., 2003; Lee et al., 2003). *LEC1* and *LEC1-like* (*L1L1*) are partially functionally redundant (Yamamoto et al., 2009), and overexpression of *GhL1L1* accelerated embryonic cell formation (Xu et al., 2019). In our study, *NF-YB9* (*LEC1*) was not expressed at 7- and 15-day induction, but became highly expressed at 30 days with uniconazole treatment. Similar expression patterns were observed in *NF-YB6* (LEC1-like) (Figure 5E). As previously reported, the middle cell layer of callus is endowed with the quiescent center (QC)-like transcriptional feature, where some marker genes including *JKD, LPR1*, and *ABCB19* were all co-expressed, while *4CL1* was highly expressed in explant vascular and callus founder cells of *Arabidopsis thaliana* (Lee et al., 1995; Welch et al., 2007; Zhai and Xu, 2021). As shown in Figure 5E, uniconazole affected the expression of these marker genes in the middle cell layer and inhibited the callus proliferation. Altogether, the differential expression of SE-related marker genes induced by uniconazole may contribute to pluripotency acquisition *via* establishing QC-like stem cell identity in callus (Zhai and Xu, 2021). In summary, we showed that uniconazole promotes the process of SE in cotton by inhibiting callus proliferation and promotes the transformation of callus into EC (Figure 6).

## Data Availability Statement

The data presented in this study are deposited in NCBI repository, accession number is PRJNA808630.

## Author Contributions

YC, XG, and FL designed the research. YC and HY performed the research. YW bred the plant materials. HY prepared samples for RNA sequencing. XG and YC analyzed the data. YC, YX, and XG wrote the manuscript. All authors have read and agreed to the published version of the manuscript.

## Conflict of Interest

The authors declare that the research was conducted in the absence of any commercial or financial relationships that could be construed as a potential conflict of interest.

## Publisher’s Note

All claims expressed in this article are solely those of the authors and do not necessarily represent those of their affiliated organizations, or those of the publisher, the editors and the reviewers. Any product that may be evaluated in this article, or claim that may be made by its manufacturer, is not guaranteed or endorsed by the publisher.

## Supplementary Material

Supplementary Figure 1 Uniconazole inhibits the growth of seedlings in *Gossypium hirsutum* L. cv ZM24. **(A)** Phenotype of non-treated control and uniconazole-treated ZM24 seedlings. **(B)** Petioles and leaves of non-treated control and uniconazole-treated ZM24 plants at the fourth leaf stage of development. **(C)** Longitudinal sections of the 4th petioles of uniconazole treated and control seedlings. Scale bar = 50 μm.

Supplementary Figure 2 Embryogenic differentiation rate of ZM24 seedling hypocotyl-derived calli cultivated on MS medium supplemented with 0–17 μM uniconazole. Differentiation of embryogenic callus (EC) was assessed after 60 days of culture. Values represent means ± SD.

Supplementary Figure 3 Phenotype of ECs after 60 days of cultivation on callus-induction medium (CK) and callus induction medium supplemented with uniconazole (Uni). Scale bar = 5 mm.

Supplementary Figure 4 Differentiation rate of callus treated with different concentrations of abscisic acid (ABA) plus uniconazole (Uni) in ZM24. After a callus culture of 60 days, the EC differentiation rate was calculated.

Supplementary Figure 5 Quantitative real-time PCR analysis of the expression patterns of differentially expressed genes in callus cultured with or without uniconazole.

Supplementary Table 1 The primers used in this study.

Supplementary Table 2 Dataset summary of the sequence alignment results of the sample sequencing data to the selected reference genome.

Supplementary Table 3 Annotation and the Fragments Per Kilobase of transcript per Million mapped reads (FPKM) values of the differentially expressed genes.
