# Supplementary figures and images for: Uniconazole Augments Abscisic Acid in Promoting Somatic Embryogenesis in Cotton (Gossypium hirsutum L.)
Source: Front Plant Sci. 2022 Apr 4;13:865778. doi: 10.3389/fpls.2022.865778 (PMC9014122; doi:10.3389/fpls.2022.865778)

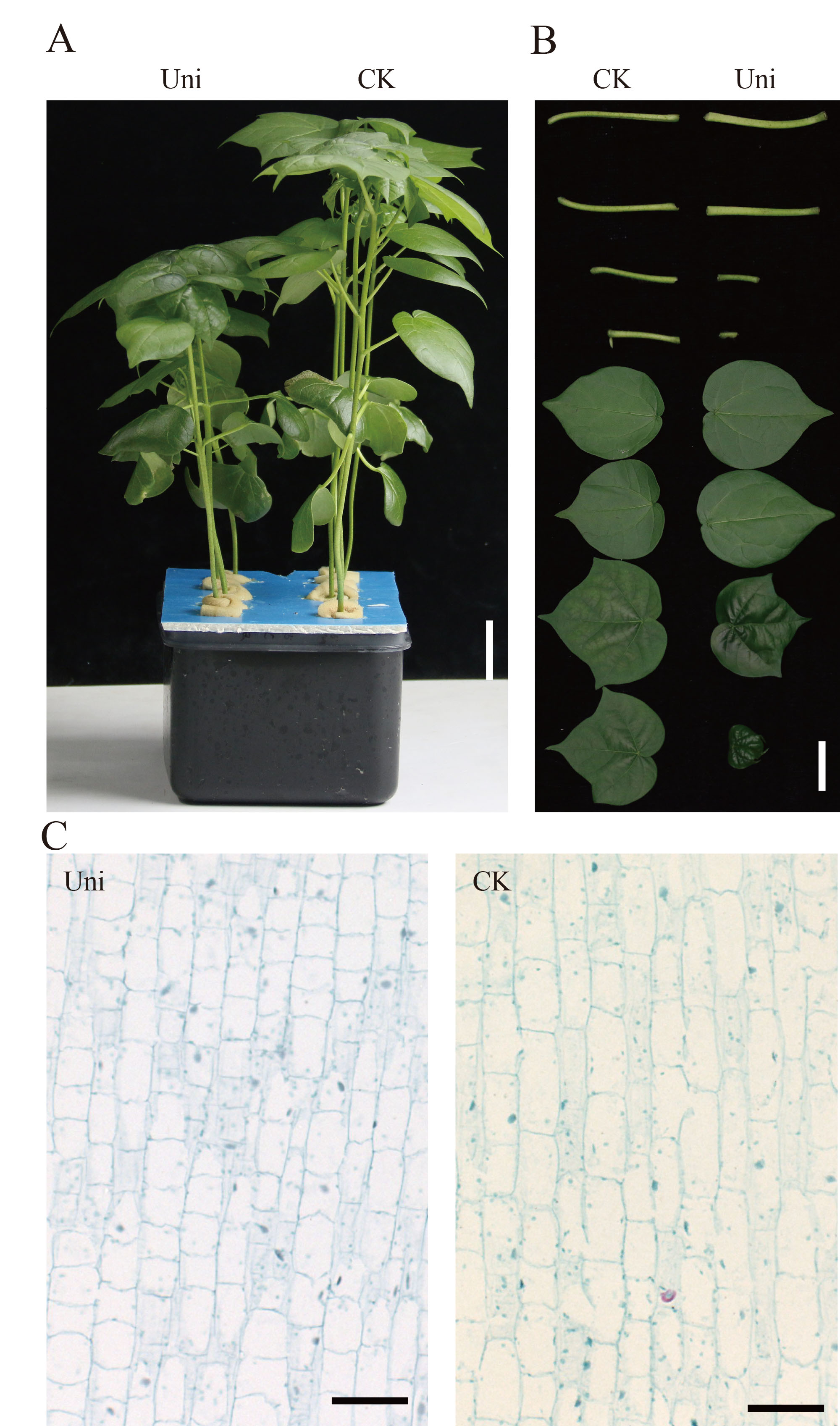

Supplement: Supplementary Figure 1 — Uniconazole inhibits the growth of seedlings in Gossypium hirsutum L. cv ZM24. (A) Phenotype of non-treated control and uniconazole-treated ZM24 seedlings. (B) Petioles and leaves of non-treated control and uniconazole-treated ZM24 plants at the fourth leaf stage of development. (C) Longitudinal sections of the 4th petioles of uniconazole treated and control seedlings. Scale bar = 50 μm. [file Image_1.JPEG]

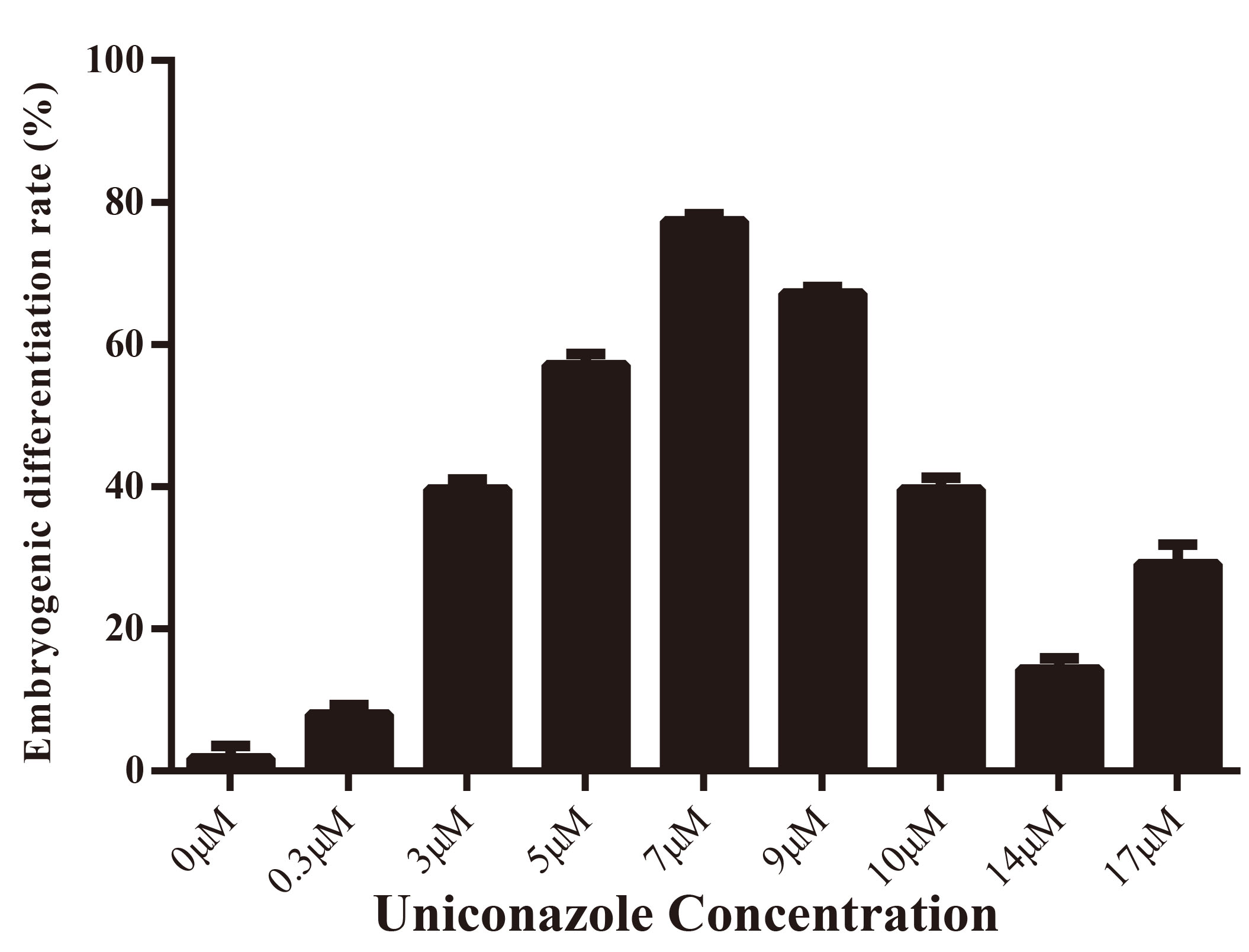

Supplement: Supplementary Figure 2 — Embryogenic differentiation rate of ZM24 seedling hypocotyl-derived calli cultivated on MS medium supplemented with 0–17 μM uniconazole. Differentiation of embryogenic callus (EC) was assessed after 60 days of culture. Values represent means ± SD. [file Image_2.JPEG]

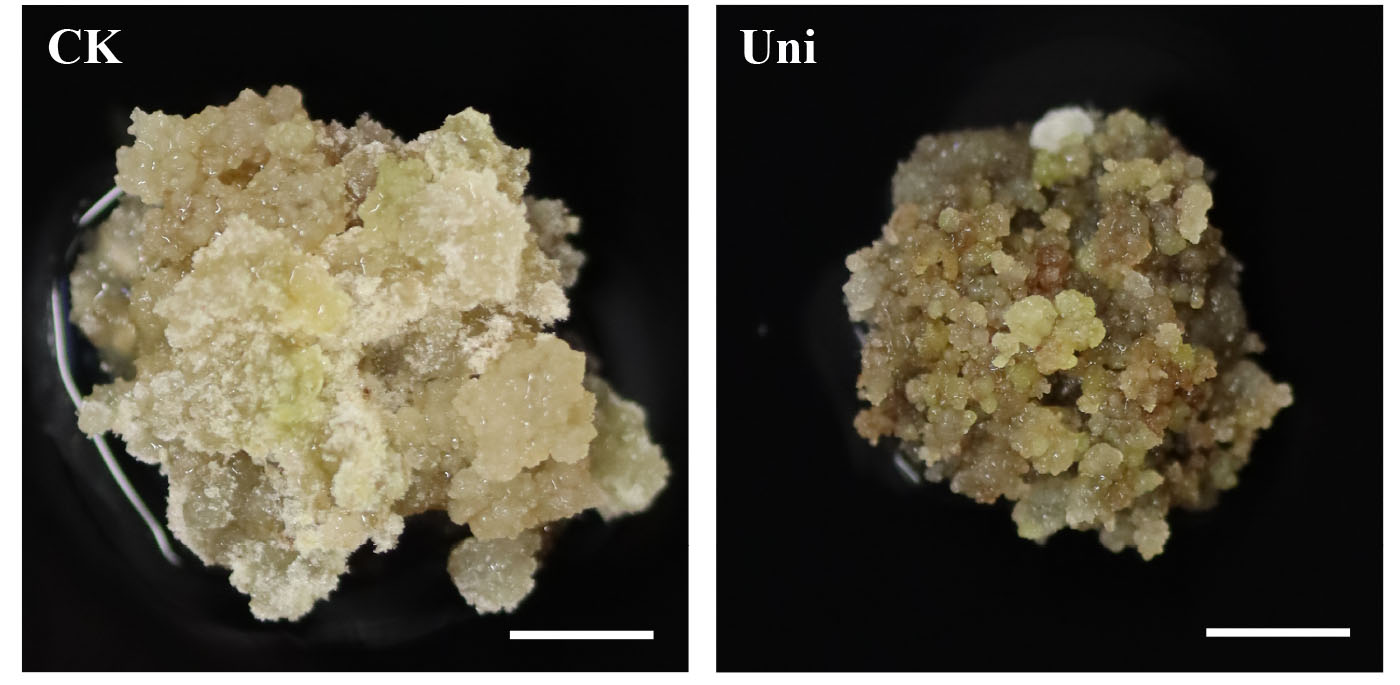

Supplement: Supplementary Figure 3 — Phenotype of ECs after 60 days of cultivation on callus-induction medium (CK) and callus induction medium supplemented with uniconazole (Uni). Scale bar = 5 mm. [file Image_3.JPEG]

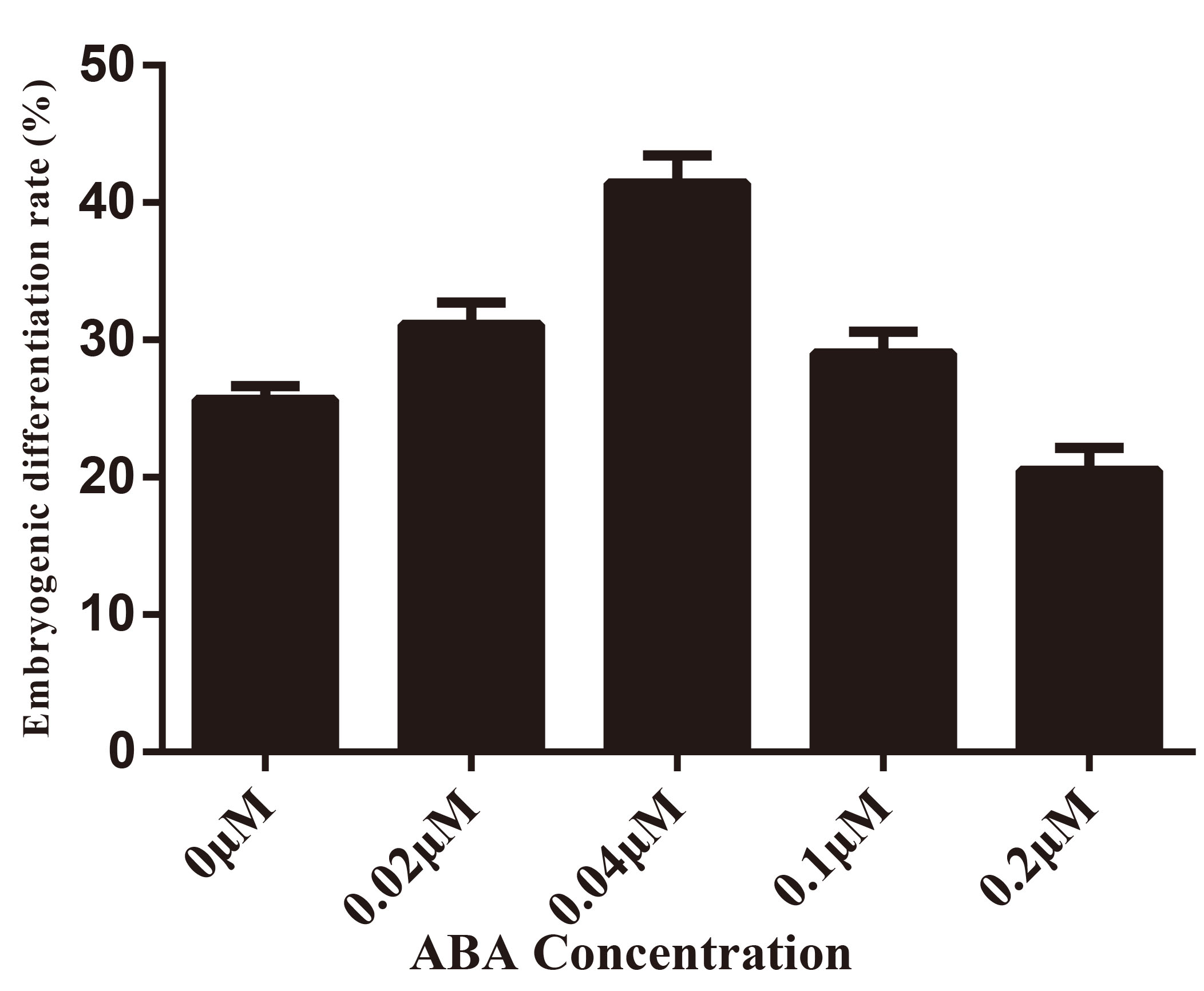

Supplement: Supplementary Figure 4 — Differentiation rate of callus treated with different concentrations of abscisic acid (ABA) plus uniconazole (Uni) in ZM24. After a callus culture of 60 days, the EC differentiation rate was calculated. [file Image_4.JPEG]

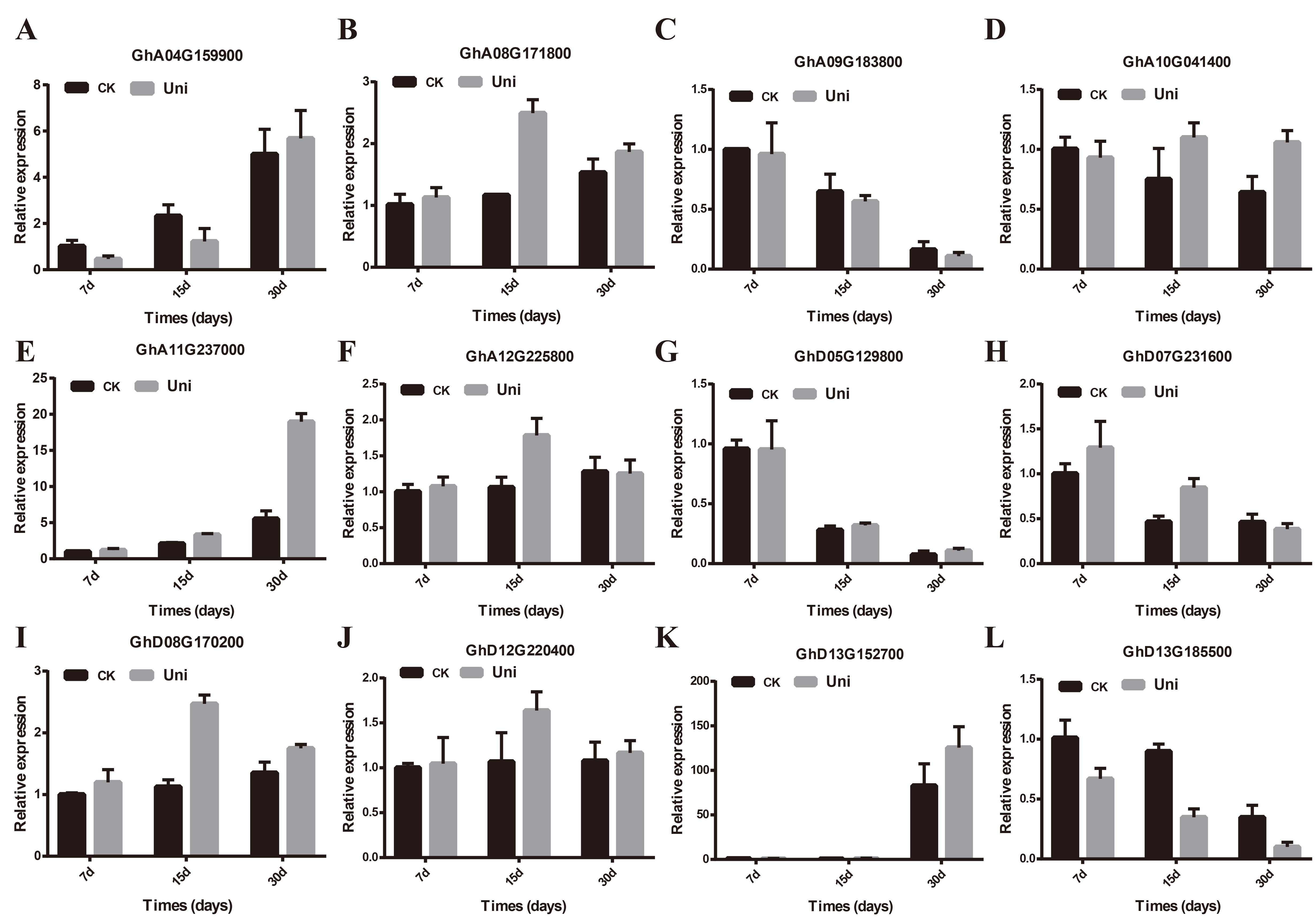

Supplement: Supplementary Figure 5 — Quantitative real-time PCR analysis of the expression patterns of differentially expressed genes in callus cultured with or without uniconazole. [file Image_5.JPEG]
